# Crystal structure of 4-bromo­cinnamic anhydride

**DOI:** 10.1107/S2056989025007789

**Published:** 2025-09-05

**Authors:** Yuliana Ramos Cotrina, Jhesua Valencia, Ronan Le Lagadec, Fernando Cuenú-Cabezas, Jovanny A. Gómez Castaño

**Affiliations:** a Laboratorio de Química Inorgánica y Catálisis Programa de Química Universidad del Quindío, Carrera 15 Calle 12 Norte Armenia 630004 Colombia; b Instituto de Química UNAM, Circuito Exterior s/n, Ciudad Universitaria, Ciudad de México 04510, Mexico; c Grupo Química-Física Molecular y Modelamiento Computacional (QUIMOL) Escuela de Ciencias Químicas Universidad Pedagógica y Tecnológica de Colombia Sede Tunja Avenida Central del Norte Tunja 150003 Boyacá Colombia; National Taras Shevchenko University of Kyiv, Ukraine

**Keywords:** crystal structure, cinnamic anhydride, supra­molecular inter­actions, O⋯π contacts

## Abstract

In the crystal, mol­ecules of the title compound [systematic name: (*E*)-3-(4-bromo­phen­yl)prop-2-enoyl (*E*)-3-(4-bromo­phen­yl)prop-2-enoate], C_18_H_12_Br_2_O_3_, reside across twofold axes passing through the central O atom of the C(O)—O—C(O) linkage [*Z*′ = ½]. The mol­ecule framework adopts an *E* configuration across the C=C bonds and a *gauche* conformation across the anhydride bridge, with a O—C—O—C torsion angle of 31.70 (11)°. The three-dimensional supra­molecular structure is governed by the inter­play of C—H⋯O hydrogen bonds and slipped stacking inter­actions involving carbon­yl/C_6_H_4_Br and anti­parallel C_6_H_4_Br/C_6_H_4_Br pairs. Hirshfeld surface and fingerprint plot analyses reveal major contributions from Br⋯H/H⋯Br and O⋯H/H⋯O contacts. The largest inter­action energies (up to −48.9 kJ mol^−1^) are associated with stacking of the mol­ecules, which highlight the dispersion-dominated stabilization. The comparable energetics of hydrogen-bonded pairs (−37.9 kJ mol^−1^) is a result of as many as four synergetic geometrically favorable C—H⋯O inter­actions. The study represents the first structural characterization of a *p*-halogenated cinnamic anhydride and these findings could be applicable to crystal design with cinnamic derivatives.

## Abstract 2

The title compound features a three-dimensional supra­molecular architecture, which is sustained by a set of weak hydrogen bonding and stacking inter­actions. Hirshfeld surface and inter­action energy analyses confirm dispersion-driven inter­actions as the dominant contributors to the packing.

## 1. Chemical context

Cinnamic anhydride derivatives, *R*—CH=CH—C(O)—O—C(O)—CH=CH—*R*′, where *R* and *R*′ denote aromatic substituents, represent a versatile class of compounds applicable to organic synthesis, medicinal chemistry, and materials science (Raja *et al.*, 2017 ▸). Their electrophilic carbonyl groups render them reactive toward nucleophiles such as alcohols, amines and enolates, enabling the selective introduction of carbonyl functionalities into diverse mol­ecular frameworks (Lin *et al.*, 2021 ▸; Robinson *et al.*, 2013 ▸). Noteworthy applications include esterification of xylans using ionic liquids to produce hemicellulose derivatives (Yang *et al.*, 2017 ▸), the efficient one-pot synthesis of thio­esters with sodium thio­sulfate penta­hydrate (Liao & Liang, 2018 ▸), and C—H activation strategies such as rhodium(I)-catalyzed alkenylation of 2-pyridones (Zhao *et al.*, 2019 ▸). Furthermore, their potential as selective acetyl­cholinesterase inhibitors has underscored their relevance in medicinal and neuropharmacological research (Giessel *et al.*, 2019 ▸).

Within this family, halogen-substituted cinnamic anhydrides are of particular inter­est because the electronic effects of halogen substituents can influence both the mol­ecular reactivity and crystal packing (Raja *et al.*, 2017 ▸). For example, crystal engineering with cinnanic acid derivatives attracts significant attention in the view of solid-state [2 + 2] cyclo­additions (Liu *et al.*, 2025 ▸). Despite this relevance, the crystal structures of *p*-halocinnamic anhydrides have not previously been reported. The presence of a halogen atom offers the possibility of halogen bonding and other directional inter­molecular inter­actions. Herein, we describe the first single-crystal X-ray diffraction study of such a species, namely the title compound *p*-bromo­cinnamic anhydride (I), providing detailed insights into its mol­ecular geometry and supra­molecular features in the solid state.
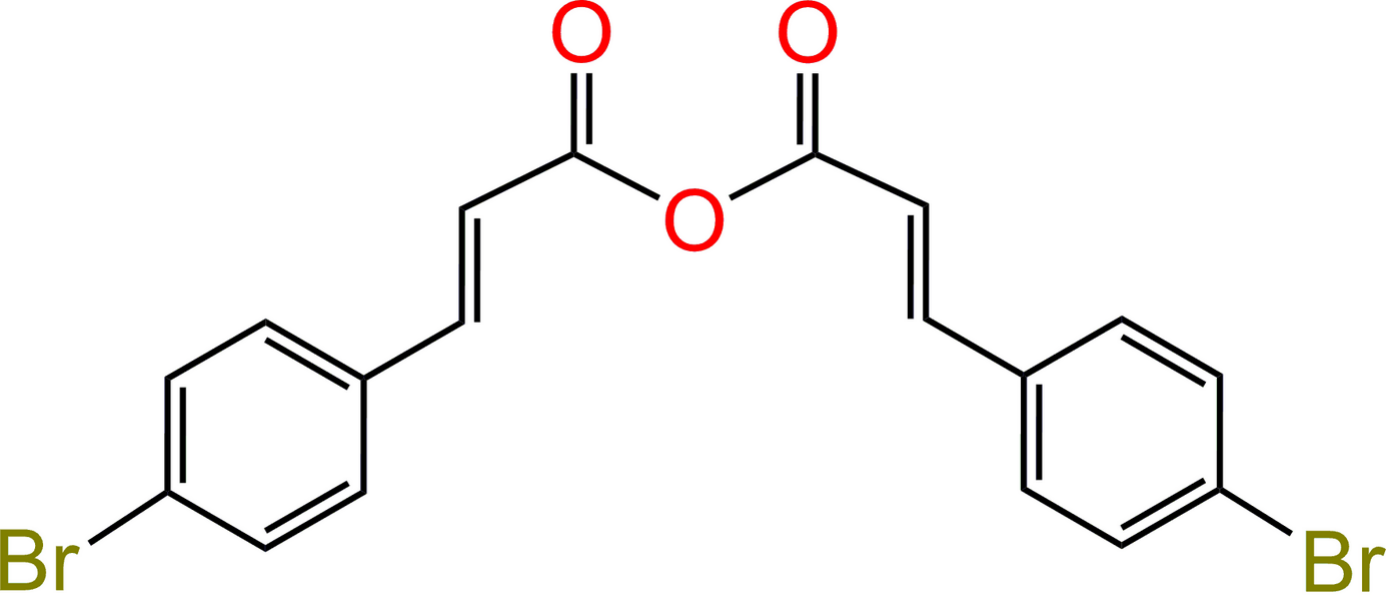


## 2. Structural commentary

**Figure 1:**
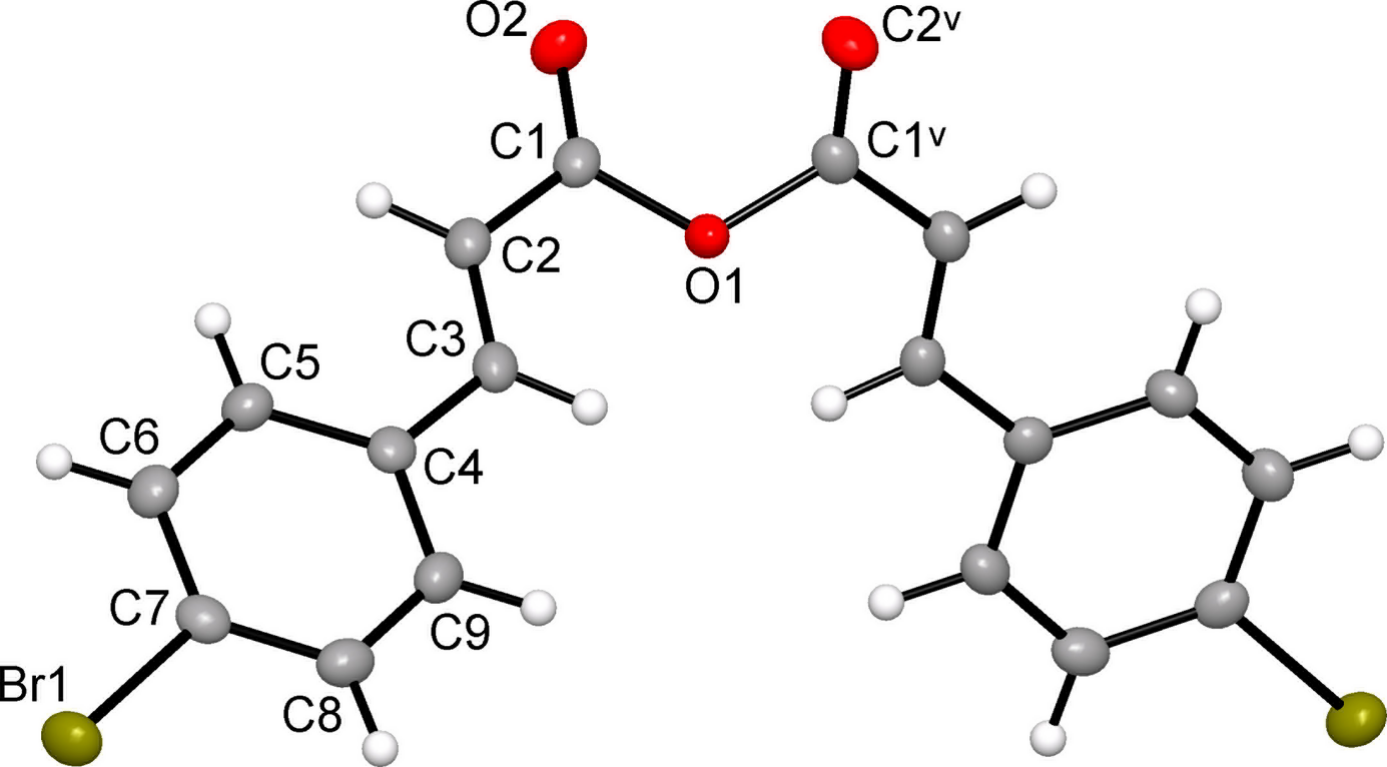
The mol­ecular structure of (I), showing the atom labeling and displacement ellipsoids drawn at the 50% probability level. [Symmetry code: (v) −*x* + 1, *y*, −*z* + 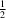.]

The title compound crystallizes in monoclinic space group *C*2/*c*, with the unique portion of the structure comprising half a mol­ecule (*Z*′ = ½) lying about a crystallographic twofold rotation axis passing through the central anhydride O1 atom (Fig. 1 ▸).

The mol­ecule adopts a *E*-configuration about each C=C double bond and displays a *gauche* conformation across the O—C—O—C anhydride bridge. The dihedral angle between the two C=C—C(O) planes is 55.36 (3)°. This conformation differs from the *syn*–*tt*–*tt* arrangement predicted to be the most stable in the gas phase for cinnamic anhydride and 3-chloro­cinnamic anhydride relatives at the B3LYP/6-311++g(d,p) level of theory (Mary *et al.*, 2014*a* ▸,*b* ▸).

Each half of the mol­ecule, comprising a 4-bromo­phen­yl–vin­yl–carboxyl fragment, is nearly planar, with dihedral angle of 4.66 (6)° between the 4-bromo­phenyl ring and the C=C—C(O) plane. This value is slightly smaller than the corresponding torsion angle of 8.85° observed in crystalline 4-bromo­cinnamic acid (Yates & Sparkes, 2013 ▸).

## 3. Supra­molecular features

**Figure 2:**
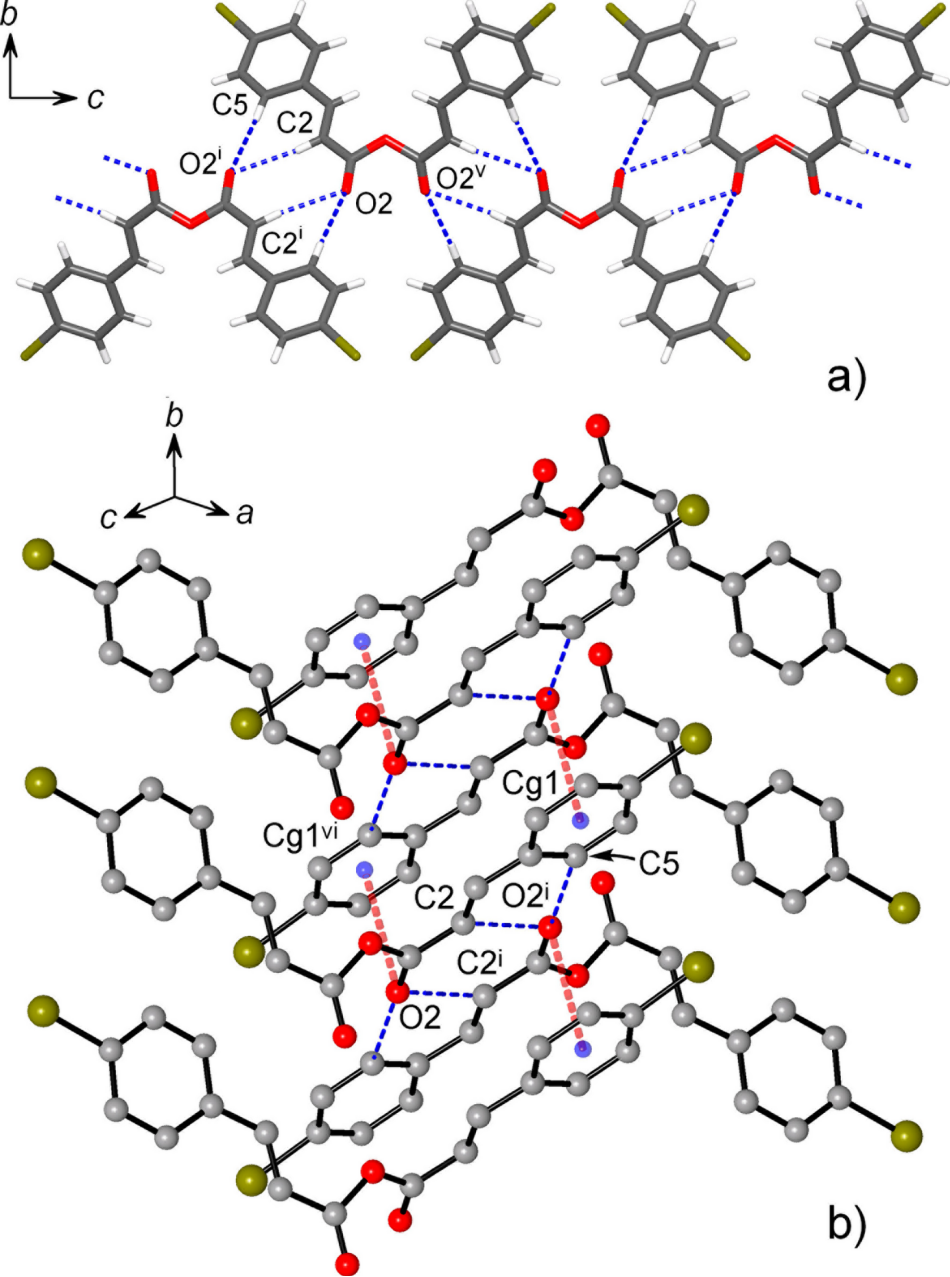
(*a*) Supra­molecular chain down the *c*-axis direction, sustained by multiple C—H⋯O hydrogen bonding; (*b*) double carbon­yl–π stacking inter­actions supporting columns along *b*-axis direction. O⋯π contacts and hydrogen bonds are indicated in red and blue, respectively. [Symmetry codes: (i) −*x* + 1, −*y*, −*z* + 1; (v) −*x* + 1, *y*, −*z* + 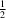; (vi) −*x* + 1, −*y* + 1, −*z* + 1.]

In the crystal, mol­ecules of (I) are linked into hydrogen-bonded chains running down the *c*-axis direction (Fig. 2 ▸), in which the inversion-related 4-bromo­phen­yl—vin­yl—carboxyl fragments [symmetry code: (i) −*x* + 1, −*y*, −*z* + 1] are linked by two pairs of reciprocal C2—H⋯O2^i^ and C5—H⋯O2^i^ hydrogen bonds (Table 1 ▸). These multiple inter­actions are geometrically favorable, as it is reflected by nearly straight angles at the hydrogen atoms [168.0 (17) and 171.6 (16)°]. The C2⋯O2^i^ separation of 3.4574 (18) Å perfectly agrees with the mean value for such hydrogen bonds from statistical analysis of cinnamate esters (3.47 Å; Pálinkó, 1999 ▸). A second type of packing-defining force is associated with two kinds of stacking inter­actions. The first motif arises from anti­parallel alignment of the inversion-related C_6_H_4_—C=C—C=O fragments, which yields double carbon­yl/ring inter­actions with notably short O2⋯*Cg*1^vi^ and O2⋯plane distances of 3.4770 (13) Å and 3.3876 (14) Å, respectively [*Cg*1 is the ring centroid; symmetry code: (vi) −*x* + 1, −*y* + 1, −*z* + 1] (Fig. 2 ▸). In combination with the above hydrogen bonding, these inter­actions assemble the mol­ecules into the layers parallel to the *bc* plane.

This pattern bears a close resemblance to the one in methyl 4-bromo­cinnamate (Leiserowitz & Schmidt, 1965 ▸). The present structure inherits not only its local hydrogen-bonding motif with multiple reciprocal inter­actions, but also stacking of hydrogen-bonded dimers leading to similar columns. In fact, (I) may be best related to methyl 4-bromo­cinnamate when considering the anhydride linkage C1—O1—C1^ii^ [symmetry code: (ii) −*x* + 1, *y*, −*z* + 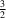] as a kind of bridge between pairs of 4-bromo­cinnamate ‘tectons’ that formally condition connection of the columns in a second dimension. This is illustrative of a general principle of crystal engineering and it may suggest certain and still unexplored potential of anhydrides for crystal design.

**Figure 3:**
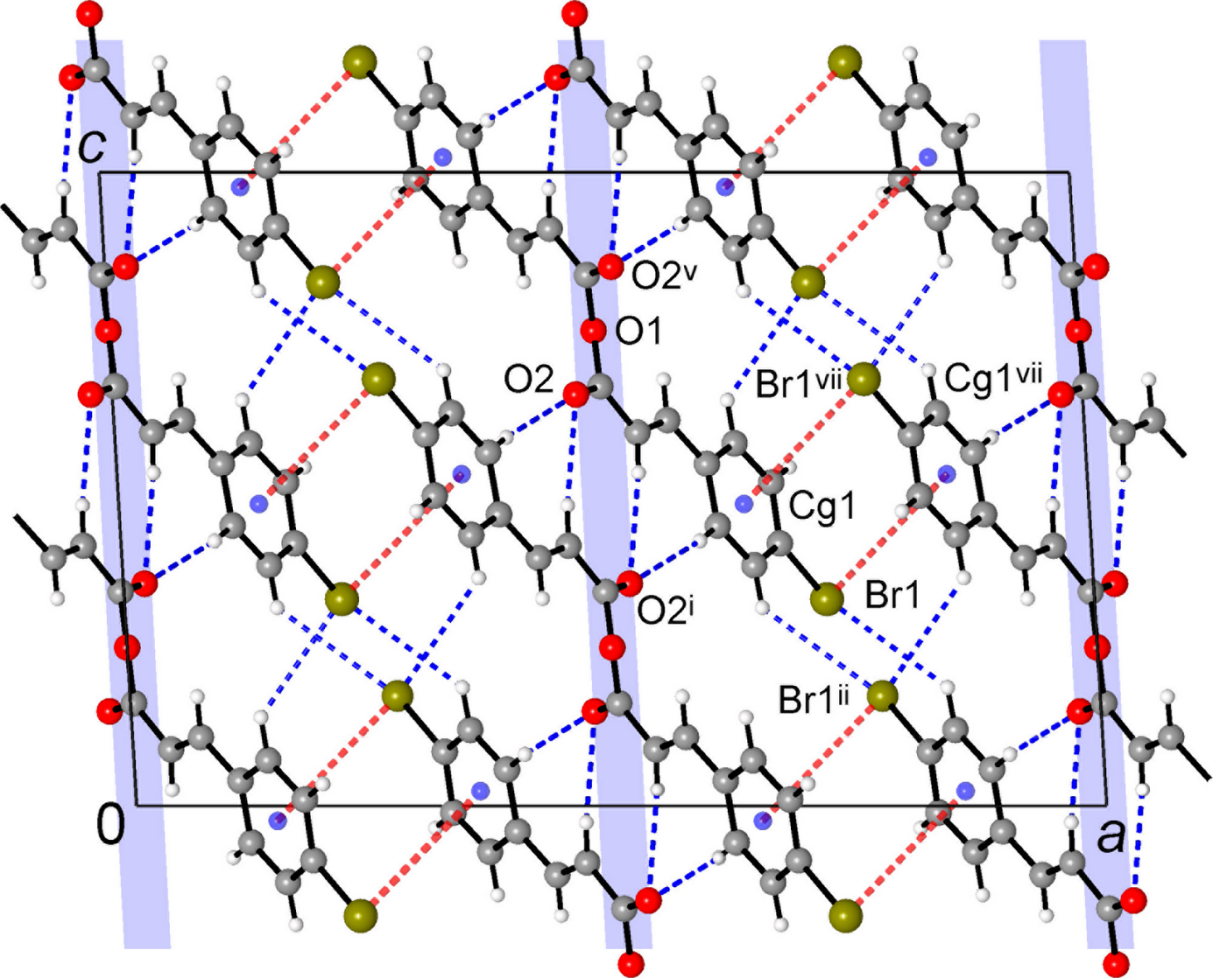
Projection of the structure in the *ac*-plane showing mutual inter­actions of C_6_H_4_Br groups and C—H⋯Br contacts between successive hydrogen- and O⋯π bonded layers (which are orthogonal to the drawing plane and are indicated by blue strips). [Symmetry codes: (i) −*x* + 1, −*y*, −*z* + 1; (ii) −*x* + 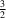, *y* − 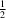, −*z* + 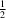; (v) −*x* + 1, *y*, −*z* + 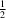; (vii) −*x* + 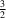, −*z* + 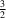, −*z* + 1.]

The second type of stacking is found between the layers (Fig. 3 ▸), in the form of slipped-anti­parallel dimers of Br—C_6_H_4_ fragments [symmetry code: (vii) −*x* + 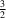, −*y* + 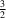, −*z* + 1], with two bromine atoms located approximately above the corresponding ring centroids [Br1⋯*Cg*1^vii^ = 4.0521 (6) Å; Br1⋯plane = 3.6963 (12) Å]. However, this stack is associated with relatively large slippage of 24.19 (2)°, defined as the angle subtended by the Br1⋯*Cg*1^vii^ axis to the ring normal. The lack of the essential overlap is also reflected by the large inter­centroid distance of 4.375 (2) Å.

The bromine atoms also engage in a set of distal contacts, *e.g.* Br1⋯Br1^viii^ = 3.7166 (2) Å [symmetry code: (viii) −*x* + 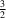, *y* + 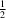, −*z* + 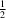]. These separations slightly exceed the sum of the van der Waals radii for bromine (3.70 Å; Bondi, 1964 ▸), suggesting a weakness of the present halogen⋯halogen contacts. As well, there are three types of H⋯Br contacts (Table 1 ▸), the shortest of which is H9⋯Br1^iv^ = 3.095 (18) Å [symmetry code: (iv) *x*, −*y* + 2, *z* + 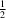]. They are reflective of very weak hydrogen bonding or dispersion forces.

## 4. Hirshfeld surface analysis

**Figure 4:**
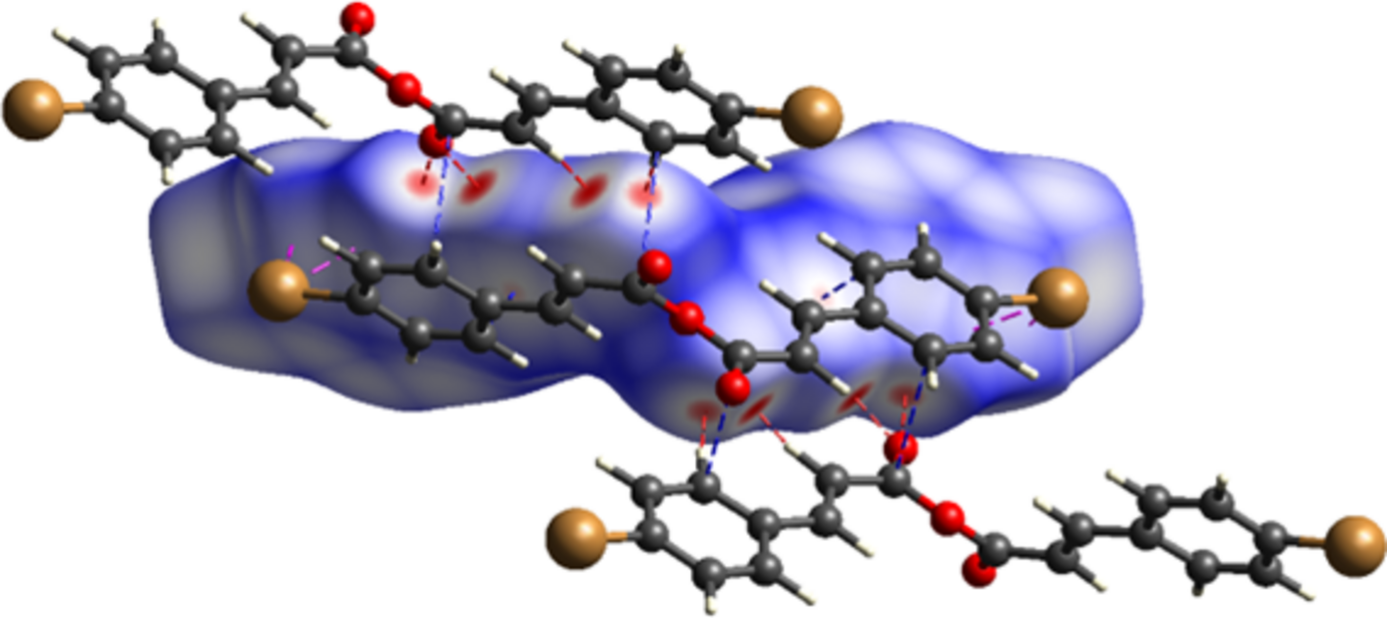
Hirshfeld surface of the mol­ecule of (I) mapped over *d*_norm_.

The Hirshfeld surface (HS) of compound (I), mapped over the normalized contact distance (*d_norm_*) (Fig. 4 ▸), highlights the contributions of carbonyl-based hydrogen bonding and C⋯C contacts associated with π–π inter­actions to the consolidation of the crystal structure (Spackman & Jayatilaka, 2008 ▸). Two sets of four intense red spots on the HS correspond to reciprocal C=O⋯H—C inter­actions, involving carbonyl oxygen atoms and two types of hydrogen donors: vinyl hydrogen (H⋯O = 2.51 Å) and aromatic *ortho*-hydrogen (H⋯O = 2.59 Å)]. Weaker red spots are associated with mutual C⋯C contacts, one set between the aromatic *ortho*-carbon and vinyl carbon (3.35 Å) along the *b*-axis, and the other between an *ortho*-carbon and a carbonyl carbon (3.38 Å) across adjacent mol­ecular *b*-axis columns.

**Figure 5:**
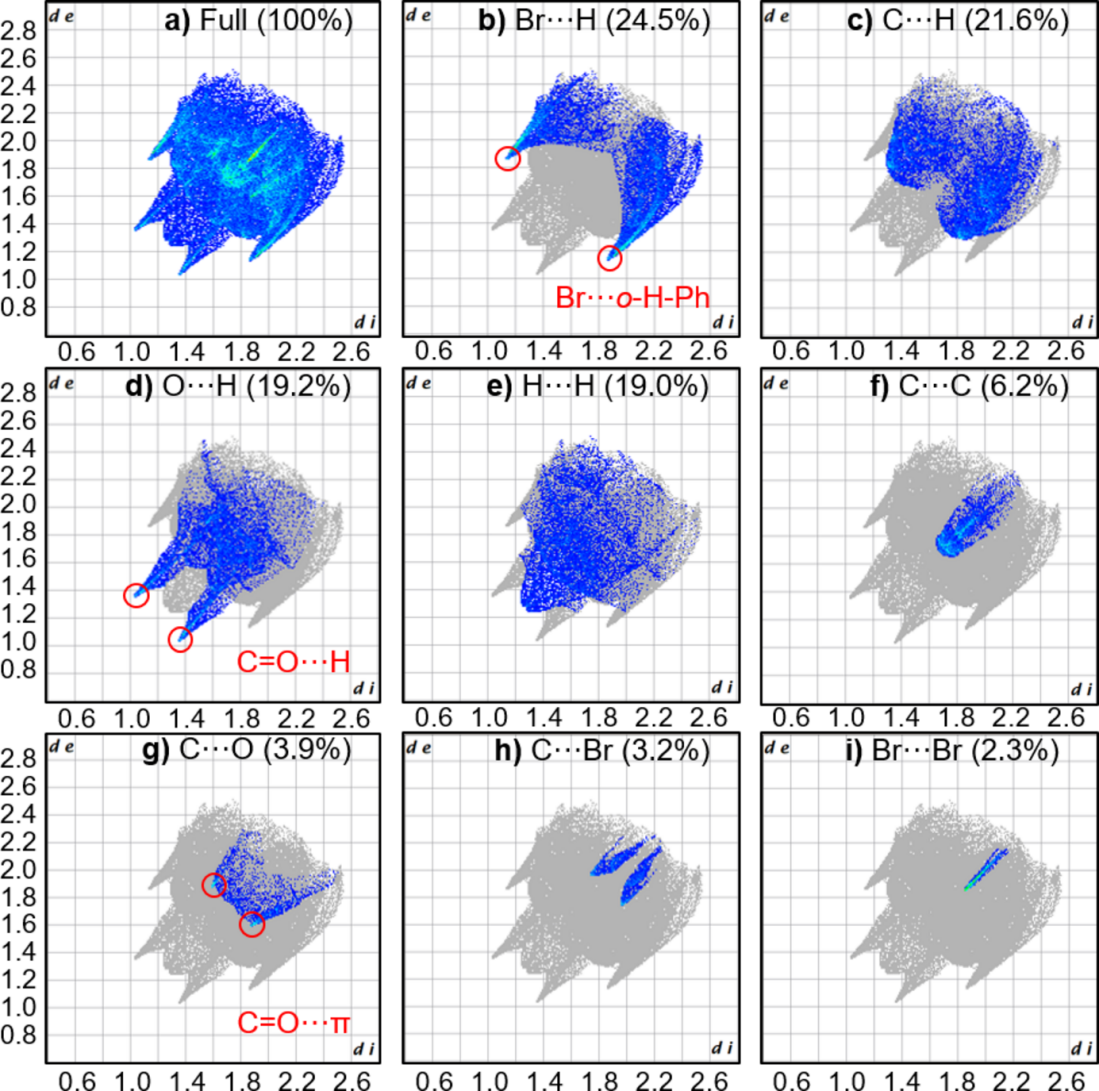
Two-dimensional fingerprint plots showing all inter­actions and delineated into the principal contributions of different types of the contacts (including also reciprocal contacts).

The two-dimensional fingerprint plots (Fig. 5 ▸) further qu­antify the contributions of specific inter­actions to the HS (McKinnon *et al.*, 2007 ▸; Spackman & McKinnon, 2002 ▸). The largest contribution arises from Br⋯H/H⋯Br contacts (24.5%), mainly involving *ortho*-positioned aromatic hydrogens. These appear as wing-like features in the fingerprint plot (Fig. 4 ▸*b*), with characteristic tips at *d*_e_/*d*_i_ ≃ 1.9/1.1 Å, indicative of directional inter­actions. Therefore, in spite of relatively large distances, the fingerprint plots allow attribution of the contacts to very weak C—H⋯Br hydrogen bonding.

O⋯H/H⋯O contacts (19.2%), corresponding to the above C=O⋯H—C inter­actions, produce the sharpest spikes at *d*_e_/*d*_i_ ≃ 1.4/1.0 Å (Fig. 5 ▸*d*). C⋯C contacts (6.2%) reflect slipped stacking between vinyl and aromatic fragments. Perceptible contributions also include C⋯O/O⋯C (3.9%) and C⋯Br/Br⋯C (3.2%), which are consistent with the observed O⋯π (*d*_e_/*d*_i_ ≃ 1.9/1.6 Å) and Br⋯π contacts. A smaller percentage arise from Br⋯Br (2.3%) contacts, corresponding to weak halogen⋯halogen inter­actions at distances approaching the sum of the van der Waals radii (3.70 Å; Bondi, 1964 ▸).

## 5. Inter­action energy calculations

**Figure 6:**
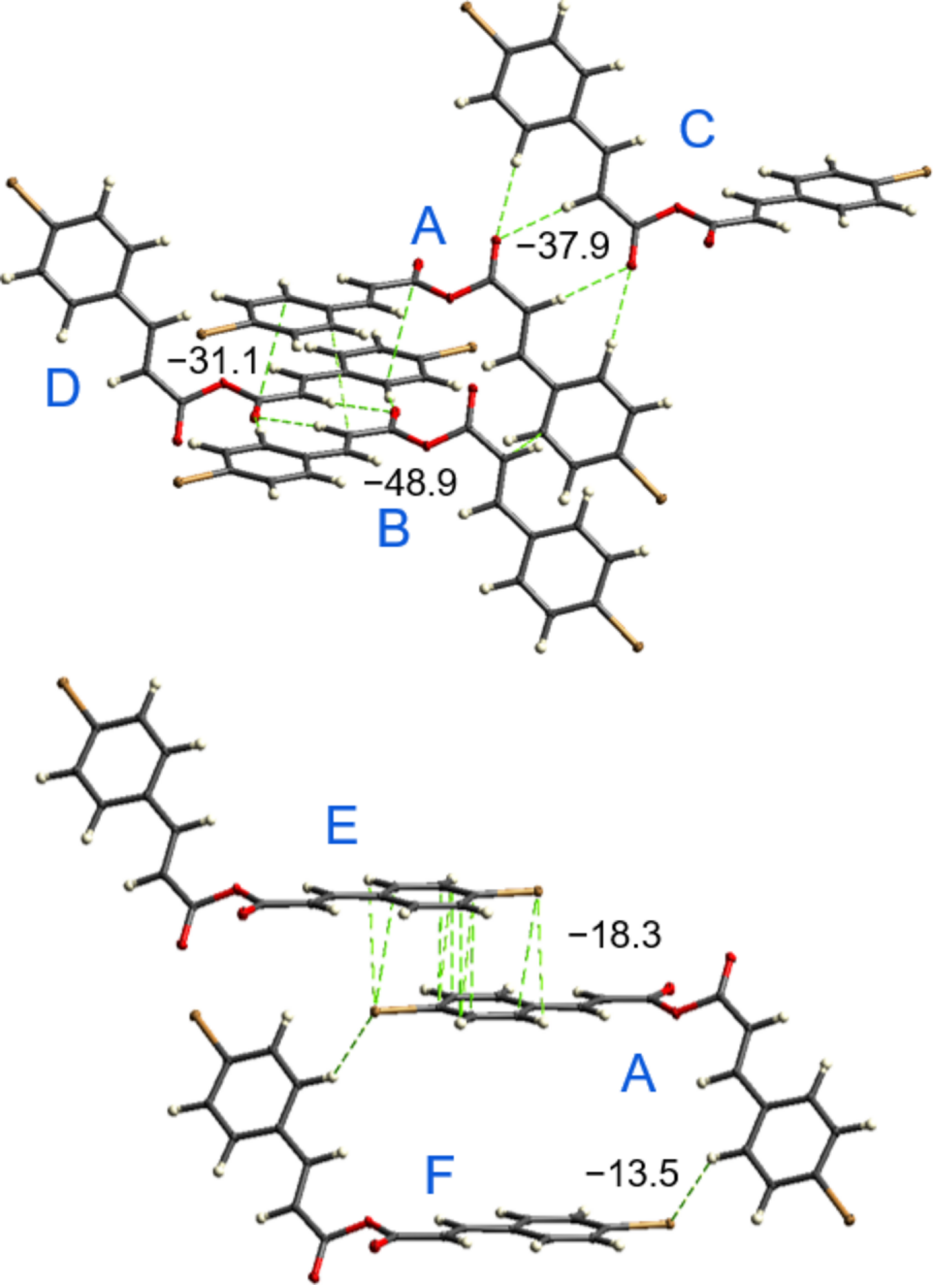
Principal pathways of pairwise inter­molecular inter­actions, identified with a cut-off limit of 12.0 kJ mol^−1^. Partial energetic contributions, symmetry operations, and geometric related parameters are summarized in Table 2 ▸.

Pairwise inter­action energies were calculated using the CE-B3LYP model implemented in *CrystalExplorer* (Mackenzie *et al.*, 2017 ▸; Turner *et al.*, 2015 ▸) to assess the energetic contributions stabilizing the supra­molecular architecture of (I) (Fig. 6 ▸). The total inter­action energy (*E*_tot_) is expressed as the sum of electrostatic (*E*_ele_), polarization (*E*_pol_), dispersion (*E*_dis_) and exchange-repulsion (*E*_rep_) terms.

Considering inter­actions with |*E*_tot_| ≥ 12.0 kJ mol^−1^, five symmetry-independent paths were identified in the closest environment of the title mol­ecule (Table 2 ▸). The strongest inter­action [*E*_tot_ = −48.9 kJ mol^−1^; *R* = 5.70 Å] occurs between translation-related mol­ecules along the *b*-axis, where C⋯C contacts between *p*-bromo­phenyl rings and vinyl groups dominate (pair *A*⋯*B*, Fig. 6 ▸). This inter­action is dispersion-driven (*E_dis_* = −58.9 kJ mol^−1^) and its significant energy originates in a relatively large inter­action area.

Second notable inter­action energy (*E*_tot_ = −37.9 kJ mol^−1^; *R* = 10.48 Å) is supported by C—H⋯O bonding of inversion-related mol­ecules (−*x* + 1, −*y*, −*z* + 1; pair *A*⋯*C*) and it is characterized by prevalence of the electrostatic component (*E_ele_* = −32.2 kJ mol^−1^). These relatively large values agree with the formation of multiple hydrogen bonds, which act in synergy. Accordingly, a comparable C—H⋯O-bonded dimer of acrylic acid, which retains only two out of four present directional bonds, revealed a lower by half inter­action energy of −19.9 kJ mol^−1^ (Czernek *et al.*, 2023 ▸). Another significant pair (*A*⋯*D*) between inversion-related mol­ecules (−*x* + 1, −*y* + 1, −*z* + 1) yields *E*_tot_ = −31.1 kJ mol^−1^ (*R* = 7.15 Å), dominated by dispersion (*E*_dis_ = −56.8 kJ mol^−1^) and arising from C=O⋯π contacts.

Two further moderate in strength inter­actions are the pair *A*⋯*E* [*E*_tot_ = −18.3 kJ mol^−1^; *R* = 12.71 Å], attributed to slipped anti­parallel stacking generating Br⋯π contacts, and pair *A*⋯*F* [*E*_tot_ = −13.5 kJ mol^−1^; *R* = 7.15 Å], associated with Br⋯H contacts. Both are governed primarily by dispersion, but in the latter case the *E*_ele_ component is also perceptible, being the third electrostatic contributor among the entire hierarchy of inter­action energies.

## 6. Database survey

A search of the Cambridge Structural Database (CSD, July 2025 release; Groom *et al.*, 2016 ▸) for cinnamic anhydride derivatives revealed no closely related crystal structures, indicating an absence of this subclass in the structural record. In contrast, numerous entries exist for cinnamic acid precursors, particularly for *trans*-cinnamic acid itself (for the most recent redetermination, see Howard *et al.*, 2009 ▸), as well as for *para*-halogenated analogues such as 4-fluoro­cinnamic (Jenkins *et al.*, 2006 ▸), 4-chloro­cinnamic (Hsieh *et al.*, 2005 ▸), and 4-bromo­cinnamic (Schmidt, 1964 ▸) acids. In addition, a recent study provided 21 examples of 4-halophenyl 4-halocinnamate esters (Liu *et al.*, 2025 ▸). The most comparable methyl 4-bromo­cinnamate (Refcode: MEBCIN; Leiserowitz & Schmidt, 1965 ▸) is mentioned above.

The absence of structurally characterized cinnamic anhydrides in the CSD may reflect intrinsic crystallization challenges associated with this subclass, including increased conformational flexibility (Mary *et al.*, 2014*a* ▸,*b* ▸), which can hinder efficient packing, and a higher propensity for hydrolysis under ambient conditions, both of which may favor amorphous or poorly crystalline forms (Raja *et al.*, 2017 ▸).

In this context, the present study reports the first single-crystal X-ray diffraction analysis of a *p*-halogenated cinnamic anhydride, providing detailed insights into its conformation, mol­ecular symmetry, and supra­molecular organization. This work establishes a useful reference point for future studies on the solid-state behavior and reactivity of cinnamic anhydride derivatives.

## 7. Synthesis and crystallization

4-Bromo­cinnamic acid was obtained from a commercial supplier and used without further purification. The title compound (I) was synthesized via a one-pot condensation reaction using *N*,*N′*-di­cyclo­hexyl­carbodi­imide (DCC) as coupling agent (Albert *et al.*, 2017 ▸). 4-Bromo­cinnamic acid (200 mg, 0.881 mmol) was dissolved in chloro­form (8 ml) and DCC (0.881 mmol) was added. The reaction mixture was refluxed for 24 h, during which time a white precipitate of di­cyclo­hexyl­urea formed, which was removed by filtration after cooling. The filtrate was concentrated under reduced pressure and the crude product was washed with cold methanol and dried affording 4-bromo­cinnamic anhydride as a colorless solid (185 mg, 95%). M.p. = 470.5–470.8 K. FT-IR (ATR, cm^−1^): 3272 (Ar CH), 2924 and 2856 (vinyl CH), 1707 (C=O), 1644 (vinyl C=C), 1599 (Ar C C), 1485 and 1447 (ring skeletal vibrations), 1367 (C—O—C), 1226 (C—O), 1069 (C—H bending), 986 (vinyl CH wag), 813 (aromatic CH bending), 618 (C—Br), 515 (C—Br stretching/ring deformation) and 488 (C—O—C bending and ring torsion).

Single crystals suitable for X-ray diffraction were obtained by slow evaporation of a dilute solution of the compound in the mixed solvents of ethyl acetate/di­chloro­methane (1:10, *v*/*v*) stored at 278 K. Colorless crystals formed over the period of 7 d.

## 8. Refinement

Crystal data, data collection and structure refinement details are summarized in Table 3 ▸. All hydrogen atoms were located and then freely refined with isotropic displacement parameters, which results in C—H = 0.94 (2)–0.971 (19) Å. One outlier (200) was omitted in the last cycles of refinement.

## Supplementary Material

Crystal structure: contains datablock(s) global, I. DOI: 10.1107/S2056989025007789/nu2012sup1.cif

Structure factors: contains datablock(s) I. DOI: 10.1107/S2056989025007789/nu2012Isup2.hkl

Supporting information file. DOI: 10.1107/S2056989025007789/nu2012Isup3.cml

CCDC reference: 2484646

Additional supporting information:  crystallographic information; 3D view; checkCIF report

Additional supporting information:  crystallographic information; 3D view; checkCIF report

## Figures and Tables

**Table 1:** Hydrogen-bond geometry (Å, °)

*D*—H⋯*A*	*D*—H	H⋯*A*	*D*⋯*A*	*D*—H⋯*A*
C2—H2⋯O2^i^	0.95 (2)	2.51 (2)	3.4574 (18)	171.6 (16)
C5—H5⋯O2^i^	0.95 (2)	2.59 (2)	3.5219 (18)	168.0 (17)
C6—H6⋯Br1^ii^	0.96 (2)	3.20 (2)	3.9492 (14)	136.0 (15)
C8—H8⋯Br1^iii^	0.94 (2)	3.20 (2)	4.1064 (15)	161.0 (15)
C9—H9⋯Br1^iv^	0.971 (19)	3.095 (18)	3.8551 (14)	136.3 (13)

Symmetry codes: (i) 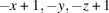; (ii) 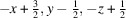; (iii) 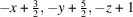; (iv) 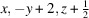.

**Table 2:** Calculated inter­action energies (kJ mol^−1^) *R* is the distance between centroids of the inter­acting mol­ecules. Inter­action energies were calculated employing the CE-B3LYP/6–31G(d,p) functional/basis set combination. The scale factors used to determine *E*_tot_: *k*_ele_ = 1.057, *k*_pol_ = 0.740, *k*_dis_ = 0.871, and *k*_rep_ = 0.618 (Mackenzie *et al.*, 2017 ▸).

Path	Symmetry relation	Type	*R* (Å)	*E*_ele_	*E*_pol_	*E*_dis_	*E*_rep_	*E*_tot_
*A*⋯*B*	*x*, *y* + 1, *z*	C⋯C; dispersion	5.70	−17.2	−5.8	−58.9	40.4	−48.9
*A*⋯*C*	-*x* + 1, −*y*, −*z* + 1	C—H⋯O; HB	10.48	−32.2	−7.7	−16.5	26.2	−37.9
*A*⋯*D*	-*x* + 1, −*y* + 1, −*z* + 1	O⋯π; dispersion	7.15	−0.7	−3.2	−56.8	32.4	−31.1
*A*⋯*E*	-*x* + 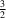 , −*y* + 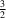 , −*z* + 1	Br⋯π stacking	12.71	6.8	−0.4	−28.9	0.0	−18.3
*A*⋯*F*	*x*, −*y* + 2, *z* + 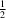	Br⋯H	7.59	−7.0	−1.1	−17.1	15.5	−13.5

**Table 3:** Experimental details

Crystal data	
Chemical formula	C_18_H_12_Br_2_O_3_
*M*_r_	436.10
Crystal system, space group	Monoclinic, *C*2/*c*
Temperature (K)	150
*a*, *b*, *c* (Å)	20.6900 (5), 5.7029 (1), 13.5534 (3)
β (°)	93.392 (1)
*V* (Å^3^)	1596.40 (6)
*Z*	4
Radiation type	Mo *K*α
μ (mm^−1^)	5.09
Crystal size (mm)	0.38 × 0.22 × 0.21

Data collection	
Diffractometer	Bruker D8 Venture κ-geometry diffractometer 208039-01
Absorption correction	Multi-scan (*SADABS*; Krause *et al.*, 2015 ▸)
*T*_min_, *T*_max_	0.505, 0.746
No. of measured, independent and observed [*I* > 2σ(*I*)] reflections	20264, 1784, 1702
*R*_int_	0.028
(sin θ/λ)_max_ (Å^−1^)	0.649

Refinement	
*R*[*F*^2^ > 2σ(*F*^2^)], *wR*(*F*^2^), *S*	0.017, 0.045, 1.09
No. of reflections	1784
No. of parameters	129
H-atom treatment	All H-atom parameters refined
Δρ_max_, Δρ_min_ (e Å^−3^)	0.27, −0.33

Computer programs: *APEX5* and *SAINT* (Bruker, 2018 ▸), *SHELXS97* (Sheldrick, 2008 ▸), *SHELXL2019/3* (Sheldrick, 2015 ▸) and *DIAMOND* (Brandenburg, 1999 ▸).

## supplementary crystallographic information

### (E)-3-(4-Bromophenyl)prop-2-enoyl (E)-3-(4-bromophenyl)prop-2-enoate Crystal data

**Table:**

C_18_H_12_Br_2_O_3_	*F*(000) = 856
*M**_r_* = 436.10	*D*_x_ = 1.814 Mg m^−^^3^
Monoclinic, *C*2/*c*	Mo *K*α radiation, λ = 0.71073 Å
*a* = 20.6900 (5) Å	Cell parameters from 9867 reflections
*b* = 5.7029 (1) Å	θ = 3.0–27.5°
*c* = 13.5534 (3) Å	µ = 5.09 mm^−^^1^
β = 93.392 (1)°	*T* = 150 K
*V* = 1596.40 (6) Å^3^	Prism, colourless
*Z* = 4	0.38 × 0.22 × 0.21 mm

### (E)-3-(4-Bromophenyl)prop-2-enoyl (E)-3-(4-bromophenyl)prop-2-enoate Data collection

**Table:**

Bruker D8 Venture κ-geometry diffractometer 208039-01	1784 independent reflections
Radiation source: micro-focus X-ray source	1702 reflections with *I* > 2σ(*I*)
Detector resolution: 52.0833 pixels mm^-1^	*R*_int_ = 0.028
ω–scans	θ_max_ = 27.5°, θ_min_ = 3.0°
Absorption correction: multi-scan (SADABS; Krause *et al.*, 2015)	*h* = −26→26
*T*_min_ = 0.505, *T*_max_ = 0.746	*k* = −7→7
20264 measured reflections	*l* = −17→17

### (E)-3-(4-Bromophenyl)prop-2-enoyl (E)-3-(4-bromophenyl)prop-2-enoate Refinement

**Table:**

Refinement on *F*^2^	Primary atom site location: structure-invariant direct methods
Least-squares matrix: full	Secondary atom site location: difference Fourier map
*R*[*F*^2^ > 2σ(*F*^2^)] = 0.017	Hydrogen site location: difference Fourier map
*wR*(*F*^2^) = 0.045	All H-atom parameters refined
*S* = 1.09	*w* = 1/[σ^2^(*F*_o_^2^) + (0.0202*P*)^2^ + 1.5368*P*] where *P* = (*F*_o_^2^ + 2*F*_c_^2^)/3
1784 reflections	(Δ/σ)_max_ = 0.002
129 parameters	Δρ_max_ = 0.27 e Å^−^^3^
0 restraints	Δρ_min_ = −0.33 e Å^−^^3^

### (E)-3-(4-Bromophenyl)prop-2-enoyl (E)-3-(4-bromophenyl)prop-2-enoate Special details

**Table:**

Geometry. All esds (except the esd in the dihedral angle between two l.s. planes) are estimated using the full covariance matrix. The cell esds are taken into account individually in the estimation of esds in distances, angles and torsion angles; correlations between esds in cell parameters are only used when they are defined by crystal symmetry. An approximate (isotropic) treatment of cell esds is used for estimating esds involving l.s. planes.

### (E)-3-(4-Bromophenyl)prop-2-enoyl (E)-3-(4-bromophenyl)prop-2-enoate Fractional atomic coordinates and isotropic or equivalent isotropic displacement parameters (Å^2^)

**Table:**

	*x*	*y*	*z*	*U*_iso_*/*U*_eq_	
Br1	0.72429 (2)	1.05887 (3)	0.32640 (2)	0.02833 (7)	
O2	0.47808 (6)	−0.05003 (19)	0.64920 (8)	0.0292 (2)	
O1	0.500000	0.2596 (2)	0.750000	0.0256 (3)	
C1	0.50364 (7)	0.1372 (3)	0.66208 (10)	0.0214 (3)	
C2	0.53902 (7)	0.2660 (2)	0.58928 (10)	0.0219 (3)	
C3	0.57054 (7)	0.4669 (3)	0.60876 (10)	0.0217 (3)	
C4	0.60848 (7)	0.5984 (2)	0.53989 (10)	0.0204 (3)	
C5	0.61593 (7)	0.5260 (3)	0.44231 (11)	0.0230 (3)	
C6	0.65139 (7)	0.6601 (3)	0.37961 (11)	0.0240 (3)	
C7	0.67923 (7)	0.8681 (3)	0.41427 (10)	0.0215 (3)	
C8	0.67399 (8)	0.9422 (3)	0.51050 (12)	0.0250 (3)	
C9	0.63846 (7)	0.8065 (3)	0.57250 (10)	0.0236 (3)	
H2	0.5376 (9)	0.194 (3)	0.5257 (14)	0.030 (4)*	
H3	0.5691 (9)	0.534 (3)	0.6727 (15)	0.027 (5)*	
H5	0.5967 (10)	0.385 (4)	0.4184 (14)	0.034 (5)*	
H6	0.6569 (10)	0.609 (4)	0.3128 (15)	0.033 (5)*	
H8	0.6933 (10)	1.082 (3)	0.5347 (15)	0.031 (5)*	
H9	0.6335 (9)	0.858 (3)	0.6399 (14)	0.028 (4)*	

### (E)-3-(4-Bromophenyl)prop-2-enoyl (E)-3-(4-bromophenyl)prop-2-enoate Atomic displacement parameters (Å^2^)

**Table:**

	*U*^11^	*U*^22^	*U*^33^	*U*^12^	*U*^13^	*U*^23^
Br1	0.02947 (10)	0.02528 (10)	0.03090 (10)	−0.00097 (6)	0.00744 (6)	0.00371 (6)
O2	0.0363 (6)	0.0248 (6)	0.0267 (5)	−0.0059 (4)	0.0044 (5)	−0.0049 (4)
O1	0.0436 (9)	0.0166 (7)	0.0171 (6)	0.000	0.0066 (6)	0.000
C1	0.0242 (7)	0.0212 (7)	0.0188 (6)	0.0041 (6)	0.0017 (5)	−0.0016 (5)
C2	0.0254 (7)	0.0222 (7)	0.0182 (6)	0.0039 (5)	0.0024 (5)	−0.0014 (5)
C3	0.0263 (7)	0.0217 (7)	0.0171 (6)	0.0050 (5)	0.0010 (5)	0.0006 (5)
C4	0.0223 (6)	0.0187 (6)	0.0201 (6)	0.0044 (5)	−0.0001 (5)	0.0008 (5)
C5	0.0267 (7)	0.0199 (7)	0.0226 (7)	0.0004 (6)	0.0018 (5)	−0.0031 (5)
C6	0.0262 (7)	0.0238 (7)	0.0224 (7)	0.0026 (6)	0.0039 (5)	−0.0028 (6)
C7	0.0194 (6)	0.0201 (6)	0.0251 (7)	0.0039 (5)	0.0022 (5)	0.0039 (5)
C8	0.0271 (7)	0.0199 (7)	0.0277 (7)	0.0003 (6)	−0.0024 (6)	−0.0017 (6)
C9	0.0285 (7)	0.0220 (7)	0.0200 (7)	0.0029 (6)	−0.0011 (5)	−0.0023 (5)

### (E)-3-(4-Bromophenyl)prop-2-enoyl (E)-3-(4-bromophenyl)prop-2-enoate Geometric parameters (Å, º)

**Table:**

Br1—C7	1.8981 (14)	C4—C5	1.403 (2)
O2—C1	1.2000 (19)	C5—C6	1.385 (2)
O1—C1^i^	1.3869 (15)	C5—H5	0.95 (2)
O1—C1	1.3869 (15)	C6—C7	1.388 (2)
C1—C2	1.461 (2)	C6—H6	0.96 (2)
C2—C3	1.337 (2)	C7—C8	1.381 (2)
C2—H2	0.95 (2)	C8—C9	1.385 (2)
C3—C4	1.462 (2)	C8—H8	0.94 (2)
C3—H3	0.95 (2)	C9—H9	0.971 (19)
C4—C9	1.399 (2)		
			
C1^i^—O1—C1	119.57 (16)	C6—C5—H5	119.1 (12)
O2—C1—O1	121.80 (13)	C4—C5—H5	120.4 (12)
O2—C1—C2	125.68 (13)	C5—C6—C7	119.23 (13)
O1—C1—C2	112.48 (12)	C5—C6—H6	120.5 (12)
C3—C2—C1	123.73 (13)	C7—C6—H6	120.2 (12)
C3—C2—H2	122.5 (11)	C8—C7—C6	121.79 (13)
C1—C2—H2	113.8 (11)	C8—C7—Br1	119.07 (11)
C2—C3—C4	125.98 (13)	C6—C7—Br1	119.14 (11)
C2—C3—H3	118.9 (11)	C7—C8—C9	118.43 (14)
C4—C3—H3	115.1 (11)	C7—C8—H8	122.2 (12)
C9—C4—C5	118.43 (13)	C9—C8—H8	119.4 (12)
C9—C4—C3	118.58 (13)	C8—C9—C4	121.58 (13)
C5—C4—C3	122.99 (13)	C8—C9—H9	119.5 (11)
C6—C5—C4	120.51 (14)	C4—C9—H9	118.9 (11)
			
C1^i^—O1—C1—O2	31.70 (11)	C4—C5—C6—C7	−0.4 (2)
C1^i^—O1—C1—C2	−150.51 (13)	C5—C6—C7—C8	1.7 (2)
O2—C1—C2—C3	−175.29 (15)	C5—C6—C7—Br1	−177.02 (11)
O1—C1—C2—C3	7.02 (19)	C6—C7—C8—C9	−1.7 (2)
C1—C2—C3—C4	177.73 (13)	Br1—C7—C8—C9	177.08 (11)
C2—C3—C4—C9	179.97 (14)	C7—C8—C9—C4	0.3 (2)
C2—C3—C4—C5	0.3 (2)	C5—C4—C9—C8	1.0 (2)
C9—C4—C5—C6	−1.0 (2)	C3—C4—C9—C8	−178.64 (13)
C3—C4—C5—C6	178.68 (13)		

Symmetry code: (i) −*x*+1, *y*, −*z*+3/2.

### (E)-3-(4-Bromophenyl)prop-2-enoyl (E)-3-(4-bromophenyl)prop-2-enoate Hydrogen-bond geometry (Å, º)

**Table:**

*D*—H···*A*	*D*—H	H···*A*	*D*···*A*	*D*—H···*A*
C2—H2···O2^ii^	0.95 (2)	2.51 (2)	3.4574 (18)	171.6 (16)
C5—H5···O2^ii^	0.95 (2)	2.59 (2)	3.5219 (18)	168.0 (17)
C6—H6···Br1^iii^	0.96 (2)	3.20 (2)	3.9492 (14)	136.0 (15)
C8—H8···Br1^iv^	0.94 (2)	3.20 (2)	4.1064 (15)	161.0 (15)
C9—H9···Br1^v^	0.971 (19)	3.095 (18)	3.8551 (14)	136.3 (13)

Symmetry codes: (ii) −*x*+1, −*y*, −*z*+1; (iii) −*x*+3/2, *y*−1/2, −*z*+1/2; (iv) −*x*+3/2, −*y*+5/2, −*z*+1; (v) *x*, −*y*+2, *z*+1/2.
